# Supplementary figures and images for: Prognostic value of the right ventricular ejection fraction using three-dimensional echocardiography: Systematic review and meta-analysis
Source: PLoS One. 2023 Jul 7;18(7):e0287924. doi: 10.1371/journal.pone.0287924 (PMC10328342; doi:10.1371/journal.pone.0287924)

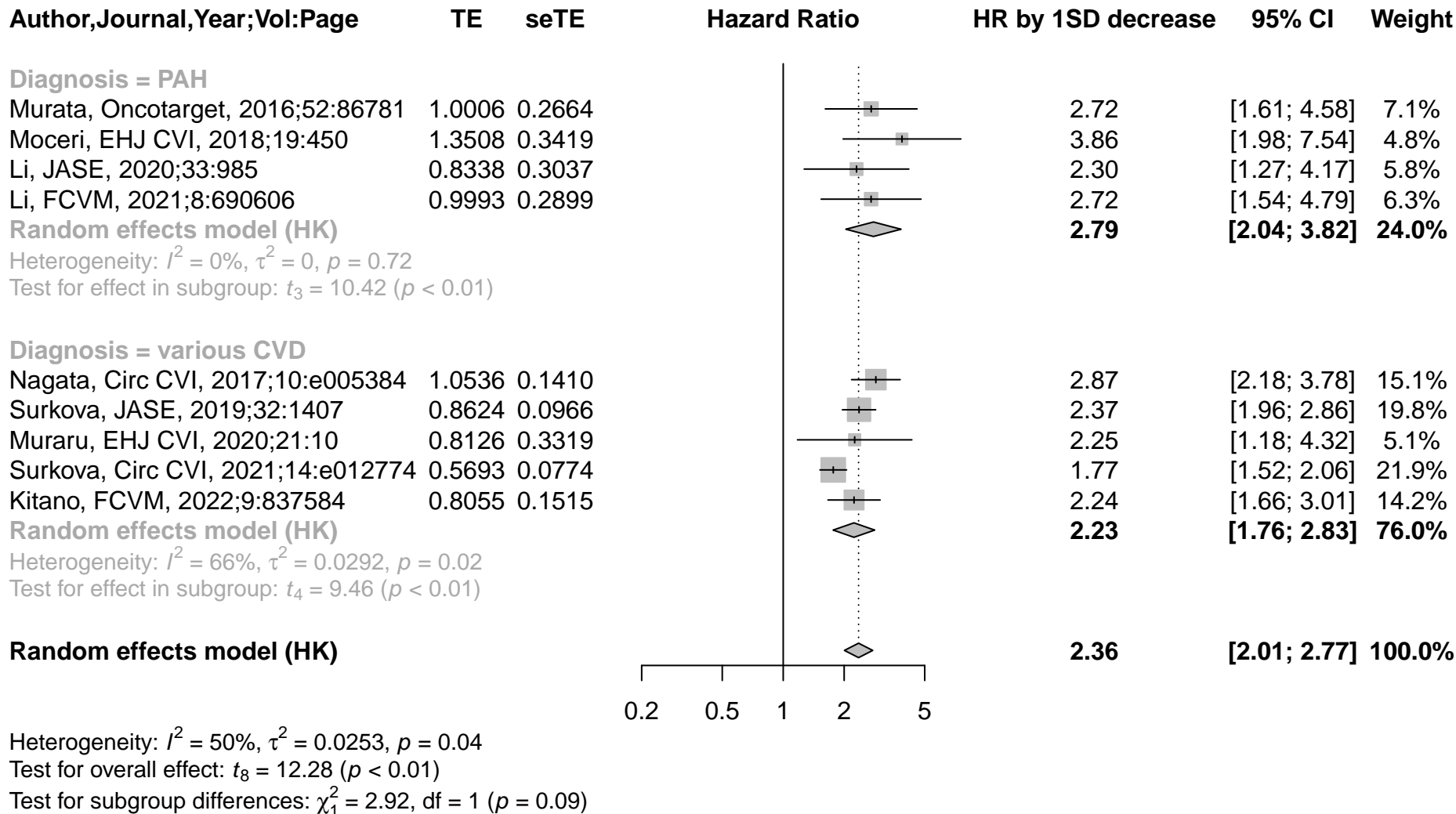

Supplement: S1 Fig — Forest plots of the hazard ratio (HR) per standard deviation (SD) reduction of right ventricular (RV) ejection fraction (EF) in patients with pulmonary arterial hypertension (upper panel) and cardiovascular disease (lower panel). CI, confidence interval; CV, cardiovascular; HR, hazard ratio; PAH, pulmonary arterial hypertension; SD, standard deviation; se, standard error; TE, treatment effect. (PDF) [file pone.0287924.s001.pdf]

A: LVEF

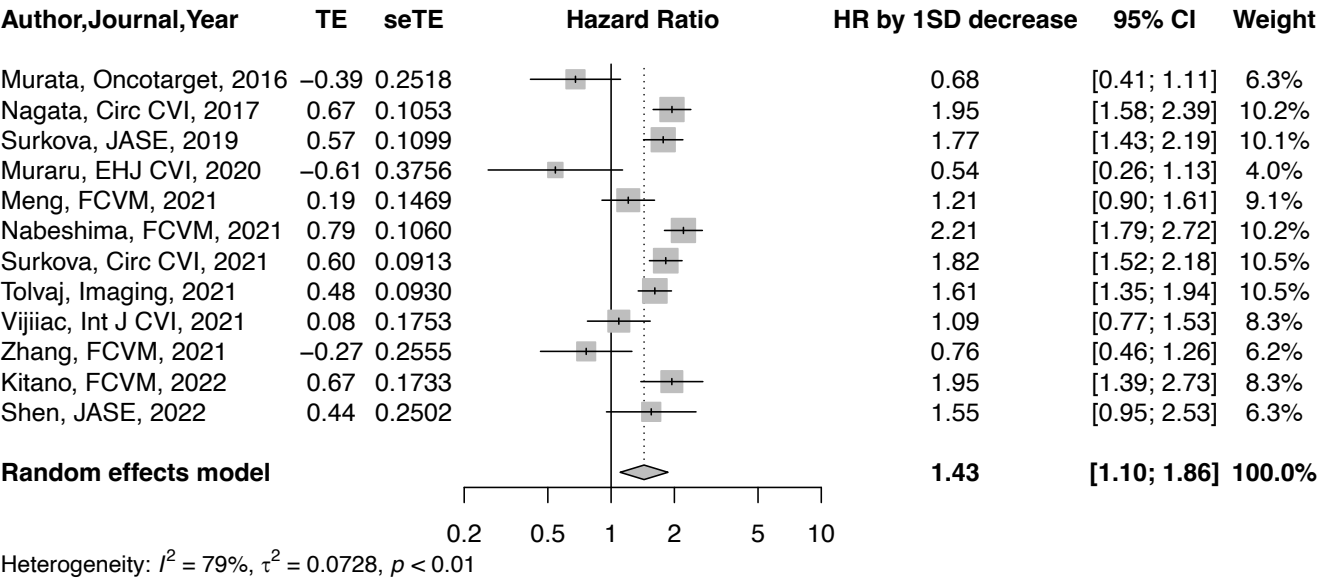

B: RVEF

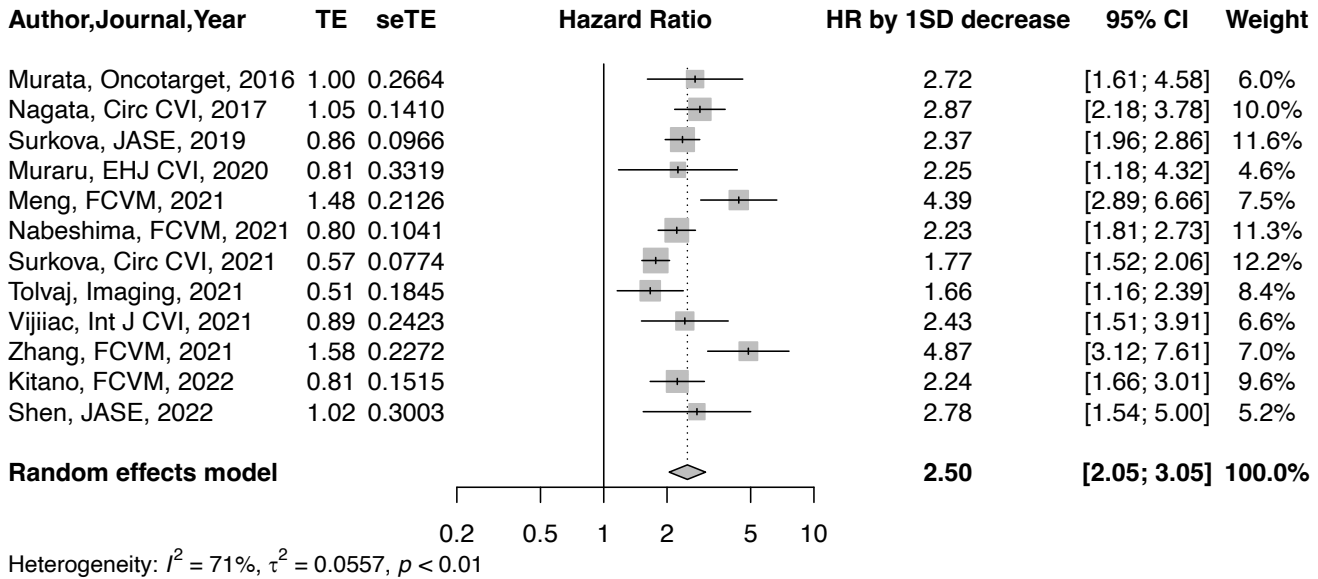

Supplement: S2 Fig — Forest plots of HR per SD reduction of left ventricular (LV) EF (A) and RVEF (B) in the same cohort from 12 studies. LVEF, left ventricular ejection fraction; RVEF, right ventricular ejection fraction. (PDF) [file pone.0287924.s002.pdf]

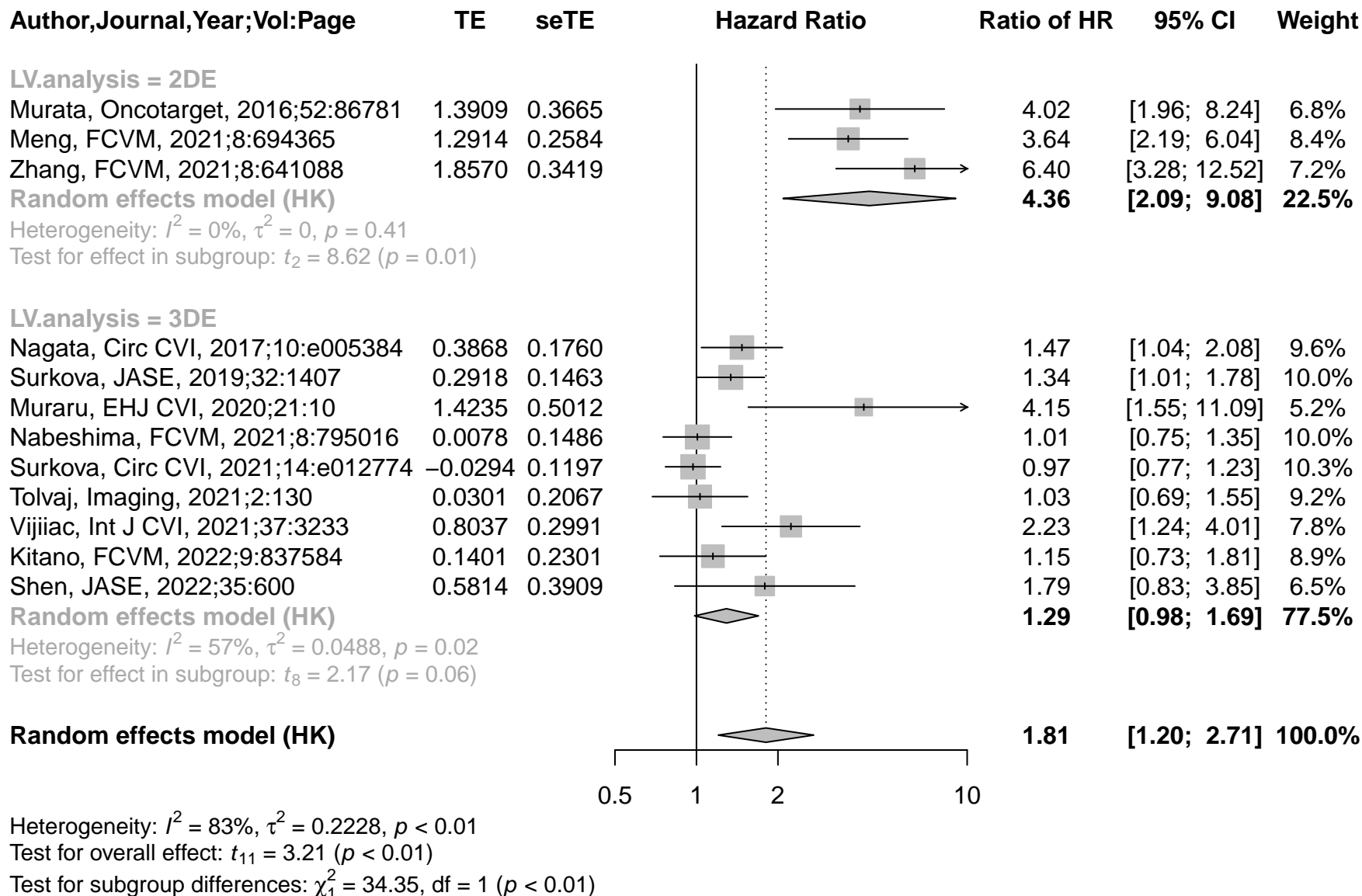

Supplement: S3 Fig — Forest plots of the ratio of HR per SD reduction between RVEF and LVEF by two-dimensional echocardiography (2DE) (upper panel) or three-dimensional echocardiography (3DE) (lower panel). 2DE, two-dimensional echocardiography; 3DE, three-dimensional echocardiography. (PDF) [file pone.0287924.s003.pdf]

A: LVGLS

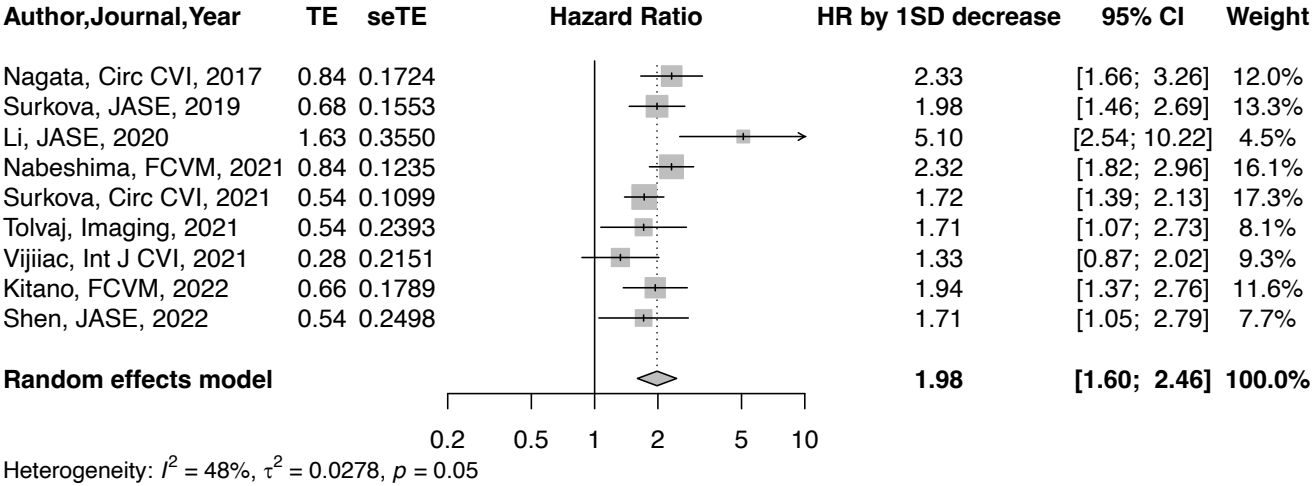

B: RVEF

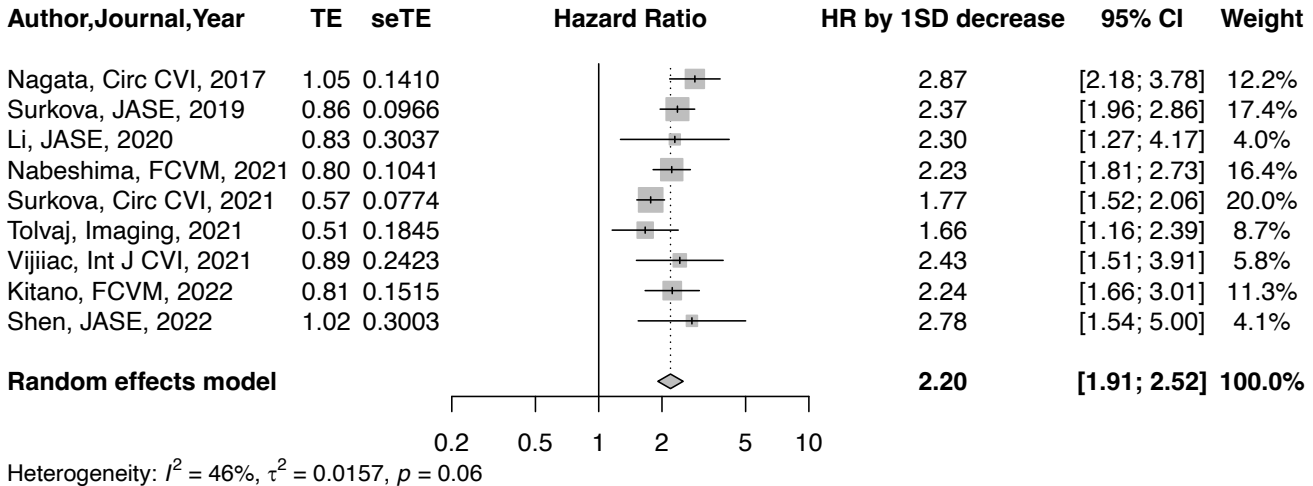

Supplement: S4 Fig — Forest plots of HR per SD reduction of LV global longitudinal strain (GLS) (A) and RVEF (B) in the same cohort from 9 studies. LVGLS, left ventricular global longitudinal strain. (PDF) [file pone.0287924.s004.pdf]

**A**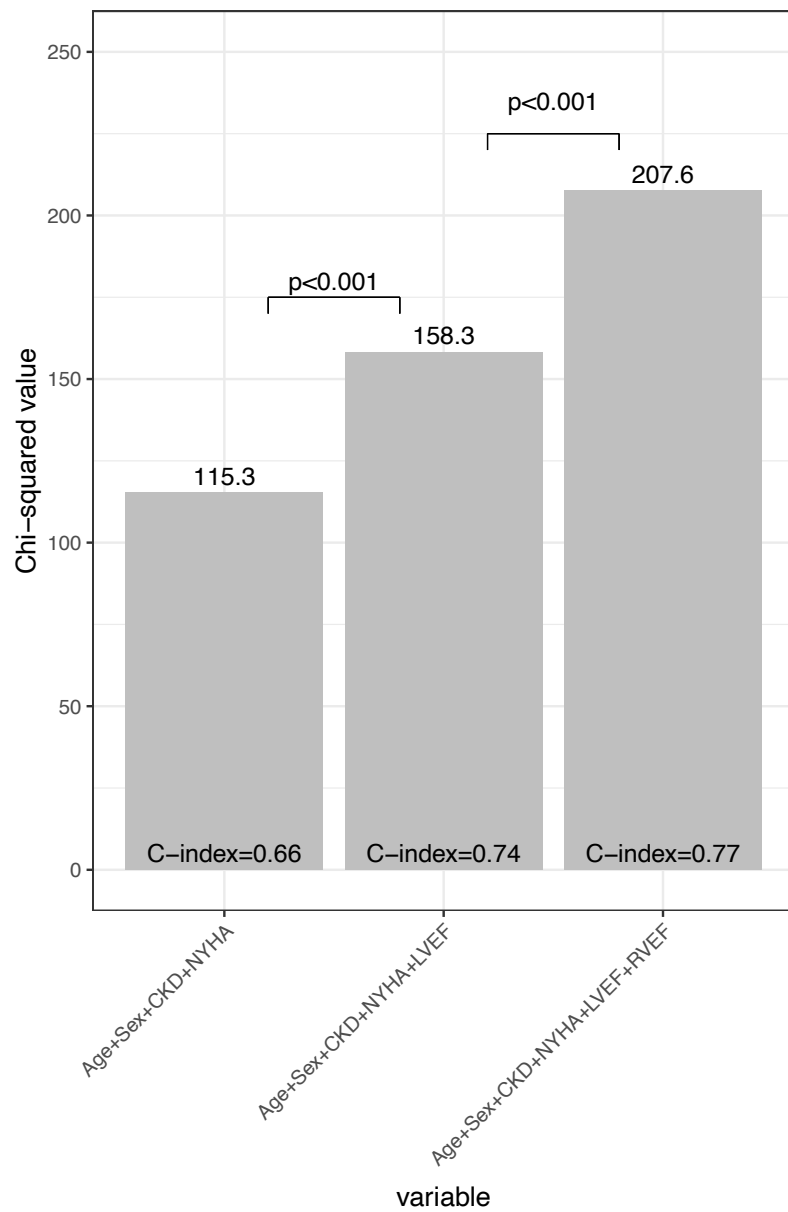**B**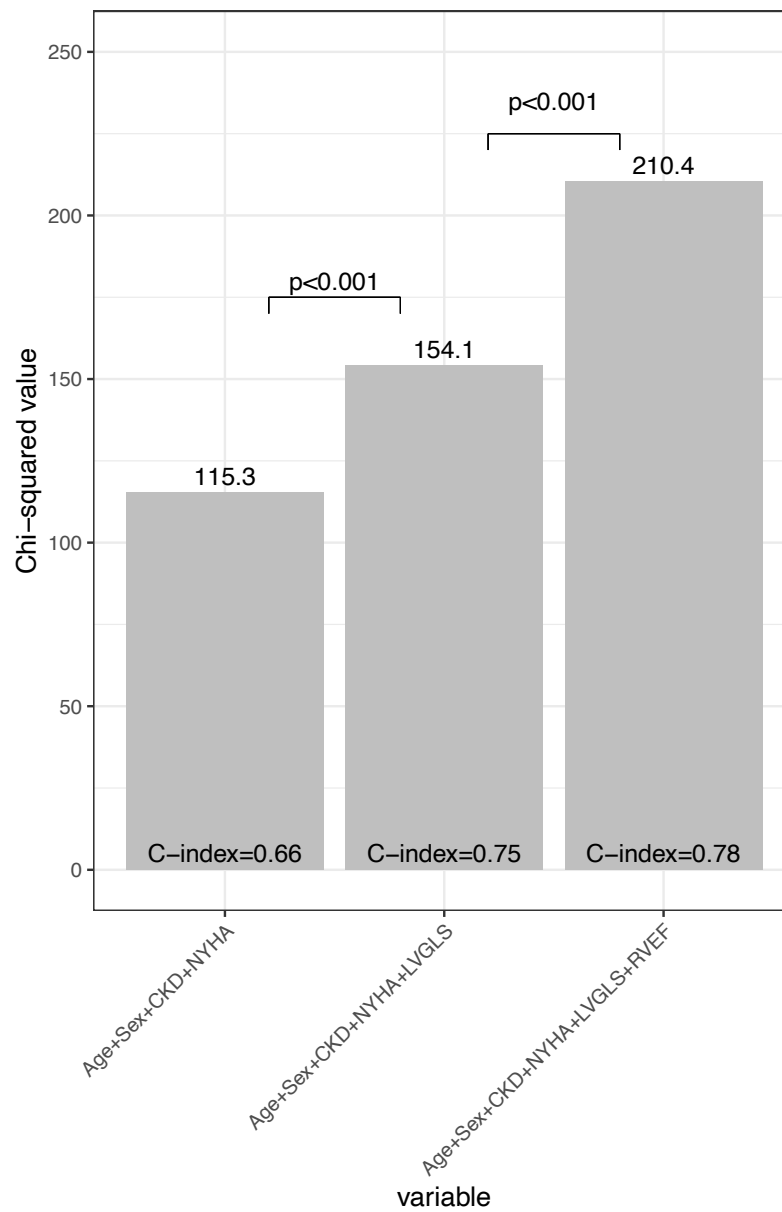

Supplement: S6 Fig — CKD, chronic kidney disease; NYHA, New York Heart Association functional class. (PDF) [file pone.0287924.s006.pdf]
