# Supplementary material for: Prognostic value of the right ventricular ejection fraction using three-dimensional echocardiography: Systematic review and meta-analysis
Source: PLoS One. 2023 Jul 7;18(7):e0287924. doi: 10.1371/journal.pone.0287924 (PMC10328342; doi:10.1371/journal.pone.0287924)
Supplement: S5 Fig — Forest plots of the ratio of HR per SD reduction between RVEF and LVGLS by 2DE (upper panel) or 3DE (lower panel). GLS, global longitudinal strain. (PDF) [file pone.0287924.s005.pdf]

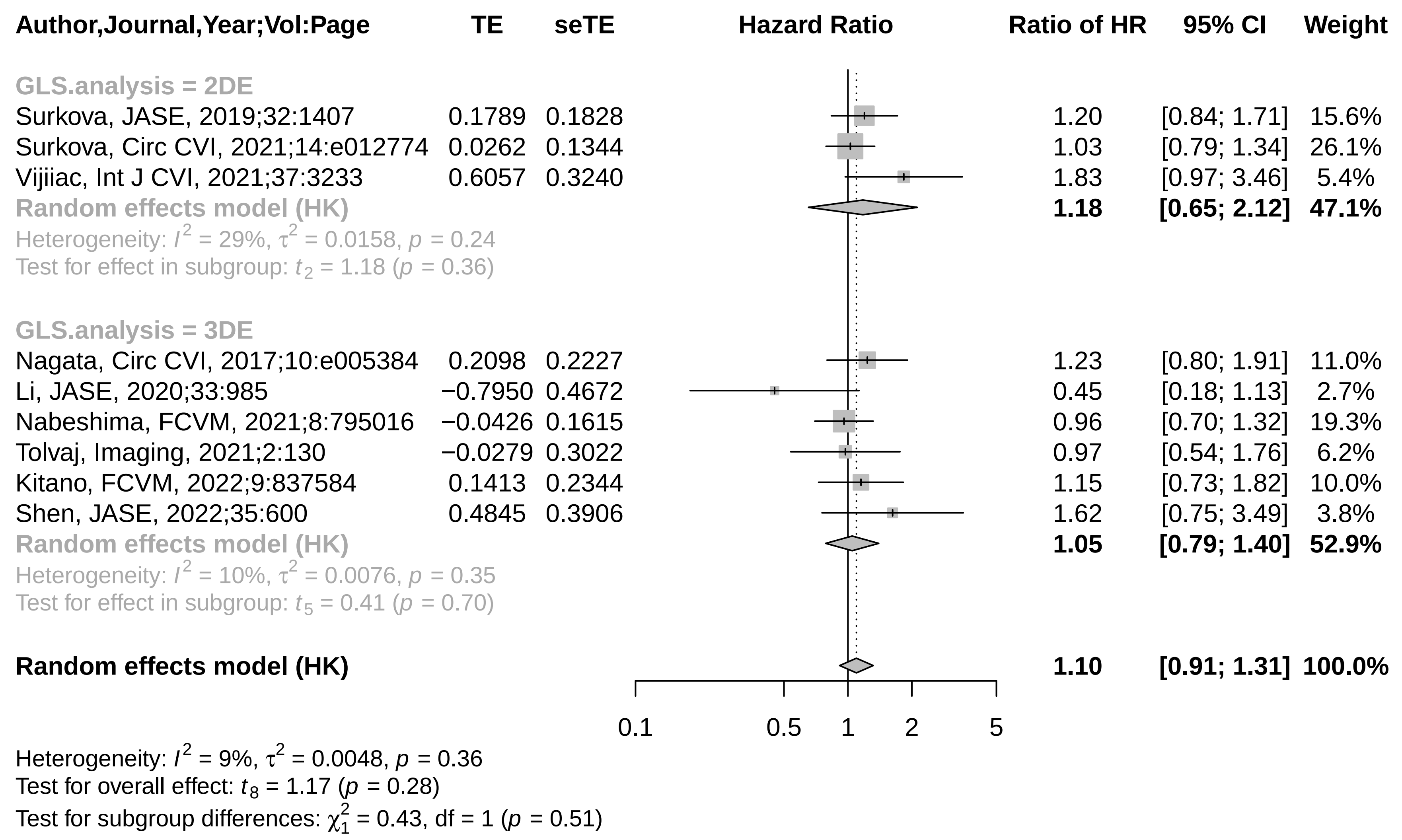
Author,Journal,Year;Vol:Page
TE
seTE
Hazard Ratio
Ratio of HR
95% CI
Weight
GLS.analysis = 2DE
Surkova, JASE, 2019;32:1407 0.1789 0.1828 1.20 [0.84; 1.71] 15.6%
Surkova, Circ CVI, 2021;14:e012774 0.0262 0.1344 1.03 [0.79; 1.34] 26.1%
Vijiiac, Int J CVI, 2021;37:3233 0.6057 0.3240 1.83 [0.97; 3.46] 5.4%
Random effects model (HK) 1.18 [0.65; 2.12] 47.1%
Heterogeneity: I^2 = 29%, τ^2 = 0.0158, p = 0.24
Test for effect in subgroup: t_2 = 1.18 (p = 0.36)
GLS.analysis = 3DE
Nagata, Circ CVI, 2017;10:e005384 0.2098 0.2227 1.23 [0.80; 1.91] 11.0%
Li, JASE, 2020;33:985 −0.7950 0.4672 0.45 [0.18; 1.13] 2.7%
Nabeshima, FCVM, 2021;8:795016 −0.0426 0.1615 0.96 [0.70; 1.32] 19.3%
Tolvaj, Imaging, 2021;2:130 −0.0279 0.3022 0.97 [0.54; 1.76] 6.2%
Kitano, FCVM, 2022;9:837584 0.1413 0.2344 1.15 [0.73; 1.82] 10.0%
Shen, JASE, 2022;35:600 0.4845 0.3906 1.62 [0.75; 3.49] 3.8%
Random effects model (HK) 1.05 [0.79; 1.40] 52.9%
Heterogeneity: I^2 = 10%, τ^2 = 0.0076, p = 0.35
Test for effect in subgroup: t_5 = 0.41 (p = 0.70)
Random effects model (HK) 1.10 [0.91; 1.31] 100.0%
0.1
0.5
1
2
5
Heterogeneity: I^2 = 9%, τ^2 = 0.0048, p = 0.36
Test for overall effect: t_8 = 1.17 (p = 0.28)
Test for subgroup differences: χ^2_1 = 0.43, df = 1 (p = 0.51)
