# Supplementary material for: Prognostic value of the right ventricular ejection fraction using three-dimensional echocardiography: Systematic review and meta-analysis
Source: PLoS One. 2023 Jul 7;18(7):e0287924. doi: 10.1371/journal.pone.0287924 (PMC10328342; doi:10.1371/journal.pone.0287924)
Supplement: S1 Table — (PDF) [file pone.0287924.s007.pdf]

Table S1: Search strategies.

I. **PubMed:**
(("prognosis"[All Fields] OR "prognostic"[All Fields] OR "prognosis"[MeSH Terms]) AND ("right ventricular ejection fraction"[All Fields] OR "RVEF"[All Fields]))
Search date on 19/May/2022
Results: 483 articles

II. **Scopus:**
TITLE-ABS-KEY (prognosis) OR TITLE-ABS-KEY (prognostic)) AND (TITLE-ABS-KEY ("right ventricular ejection fraction") OR TITLE-ABS-KEY (rvef)
Search date on 19/May/2022
Results: 349 articles

III. **Embase:**
TITLE-ABS-KEY (prognosis) OR TITLE-ABS-KEY (prognostic)) AND (TITLE-ABS-KEY ("right ventricular ejection fraction") OR TITLE-ABS-KEY (rvef)
Search date on 19/ May/ 2022
Results: 751 articles
