# Supplementary material for: Prognostic value of the right ventricular ejection fraction using three-dimensional echocardiography: Systematic review and meta-analysis
Source: PLoS One. 2023 Jul 7;18(7):e0287924. doi: 10.1371/journal.pone.0287924 (PMC10328342; doi:10.1371/journal.pone.0287924)
Supplement: S2 Table — (PDF) [file pone.0287924.s008.pdf]

Table S2: Reasons for full-text exclusion.

First Author	Year	Reason for exclusion
**La Vecchia**	2006	RVEF measured by other than 3DE
**Jensen**	2010	Not including HR of any parameter
**Meyer**	2010	RVEF measured by other than 3DE
**De Groote**	2012	RVEF measured by other than 3DE
**Meyer**	2012	RVEF measured by other than 3DE
**Desai**	2013	RVEF measured by other than 3DE
**Selimovic**	2013	RVEF measured by other than 3DE
**Cho**	2014	Not including HR of RVEF as a continuous variable, only binary variable
**Doesch**	2014	Not including HR of RVEF as a continuous variable, only binary variable
**Murninkas**	2014	Not available value of HR for RVEF
**Park**	2014	Not including HR of RVEF as a continuous variable, only binary variable
**Smith**	2014	Not including HR of RVEF as a continuous variable, only binary variable
**Courand**	2015	RVEF measured by other than 3DE
**Haghikia**	2015	Not including HR of any parameter
**Lella**	2015	Not including HR of RVEF as a continuous variable, only binary variable
**Ryo**	2015	Including HRs of some parameters, but not of RVEF
**Vanderpool**	2015	Not available value of HR for RVEF
**Abualsaud**	2016	Data including children
**De Siqueira**	2016	Not including univariate HR of RVEF, only multivariate HR
**Zamfir**	2016	Not including HR of any parameter
**da Costa Junior**	2017	Including HRs of some parameters, but not of RVEF
**Rodrigues**	2017	Not including univariate HR of RVEF, only multivariate HR
**Geva**	2018	Data including children
**Magunia**	2018	Not including HR of any parameter
**Nochioka**	2018	Not including univariate HR of RVEF, only multivariate HR
**Gill**	2019	Not including HR of any parameter
**Gumu**	2019	Not including HR of any parameter
**Diller**	2020	Data including children
**Liu**	2020	Not including HR of any parameter
**Winkelhorst**	2020	RVEF measured by other than 3DE
**Asano**	2021	Including HRs of some parameters, but not of RVEF
**Ashcroft**	2021	Not including HR of any parameter
**Kanagala**	2021	Including HR as Log RVEF
**Li**	2021	Not available exact value of HR
**Namisaki**	2021	Including patients overlap with Kitano's article.
**Spieker**	2021	Not including HR of RVEF as a continuous variable, only binary variable
**Alandejani**	2022	Not including HR of any parameter
**Bourfiss**	2022	Including HRs of strain parameters, but not of RVEF
**Li**	2022	Including HRs of some parameters, but not of RVEF
**Vijiac**	2022	Including HRs of some parameters, but not of RVEF

3DE, three-dimensional echocardiography; HR, hazard ratio; RVEF, right ventricular ejection fraction.
