# Supplementary material for: Prognostic value of the right ventricular ejection fraction using three-dimensional echocardiography: Systematic review and meta-analysis
Source: PLoS One. 2023 Jul 7;18(7):e0287924. doi: 10.1371/journal.pone.0287924 (PMC10328342; doi:10.1371/journal.pone.0287924)
Supplement: S3 Table — (PDF) [file pone.0287924.s009.pdf]

Table S3: Summary of studies quality analysis.

	Reporting				External/Internal validity				
**First author**	**(1)**	**(2)**	**(3)**	**(4)**	**(5)**	**(6)**	**(7)**	**(8)**	**(9)**
**Murata**	Yes	Yes	Yes	Yes	Yes	NR	Yes	Yes	Yes
**Nagata**	Yes	Yes	Yes	Yes	Yes	NR	Yes	Yes	Yes
**Moceri**	Yes	Yes	Yes	Yes	Yes	NR	Yes	Yes	Yes
**Surkova**	Yes	Yes	Yes	Yes	Yes	NR	Yes	Yes	Yes
**Li**	Yes	Yes	Yes	Yes	Yes	Yes	Yes	Yes	Yes
**Muraru**	Yes	Yes	Yes	Yes	Yes	Yes	Yes	Yes	Yes
**Li**	Yes	Yes	Yes	Yes	Yes	NR	Yes	Yes	Yes
**Meng**	Yes	Yes	Yes	Yes	Yes	NR	Yes	Yes	Yes
**Nabeshima**	Yes	Yes	Yes	Yes	Yes	NR	Yes	Yes	No
**Surkova**	Yes	Yes	Yes	Yes	Yes	Yes	Yes	Yes	Yes
**Tolvaj**	Yes	Yes	Yes	Yes	Yes	NR	Yes	Yes	No
**Vijiiac**	Yes	Yes	Yes	Yes	Yes	Yes	Yes	Yes	Yes
**Zhang**	Yes	Yes	Yes	Yes	Yes	NR	Yes	Yes	Yes
**Kitano**	Yes	Yes	Yes	Yes	Yes	NR	Yes	Yes	Yes
**Shen**	Yes	Yes	Yes	Yes	Yes	NR	Yes	Yes	Yes

(1), hypothesis/aim/objective described; (2), outcome described; (3), characteristics described; (4), main findings described; (5), representative population; (6), blinded to outcome data; (7), 3DE imaging protocol; (8), appropriate statistical analysis; (9), reproducibility analysis performed; NR, not reported.
