# Supplementary material for: Prognostic value of the right ventricular ejection fraction using three-dimensional echocardiography: Systematic review and meta-analysis
Source: PLoS One. 2023 Jul 7;18(7):e0287924. doi: 10.1371/journal.pone.0287924 (PMC10328342; doi:10.1371/journal.pone.0287924)
Supplement: S4 Table — Multivariable Cox proportional hazards analysis of (A) LVEF < 45%, (B) LVEF ≥ 45%, (C) LVGLS < 16%, (D) LVGLS ≥ 16% for cardiac events in validation study. (PDF) [file pone.0287924.s010.pdf]

Table S4: Multivariate Cox proportional hazards analysis of (A) LVEF < 45%, (B) LVEF ≥ 45%, (C) LVGLS < 16%, (D) LVGLS ≥ 16% for cardiac events in validation study.

A. LVEF < 45%

	N	HR (95% CI)	P value
Age (year)	417	1.01 (1.00 to 1.03)	0.12
Sex (male)	417	0.61 (0.42 to 0.89)	0.010
NYHA (Class II)	417	0.79 (0.51 to 1.23)	0.3
NYHA (Class III/IV)	417	1.40 (0.82 to 2.38)	0.2
CKD	417	1.77 (1.19 to 2.63)	0.005
LVEF (%)	417	1.01 (0.99 to 1.03)	0.4
RVEF (%)	417	0.95 (0.93 to 0.97)	<0.001

B. LVEF ≥ 45%

	N	HR (95% CI)	P value
Age (year)	723	1.04 (1.01 to 1.07)	0.002
Sex (male)	723	1.30 (0.79 to 2.14)	0.3
NYHA (Class II)	723	2.38 (1.31 to 4.32)	0.004
NYHA (Class III/IV)	723	2.71 (1.18 to 6.21)	0.019
CKD	723	2.13 (1.28 to 3.54)	0.004
LVEF (%)	723	0.99 (0.94 to 1.04)	0.7
RVEF (%)	723	0.91 (0.88 to 0.93)	<0.001

C. LVGLS < 16%

	N	HR (95% CI)	P value
Age (year)	741	1.02 (1.01 to 1.04)	0.001
Sex (male)	741	0.67 (0.48 to 0.93)	0.017
NYHA (Class II)	741	0.92 (0.62 to 1.36)	0.7
NYHA (Class III/IV)	741	1.41 (0.87 to 2.30)	0.2
CKD	741	1.85 (1.31 to 2.62)	<0.001
LVGLS (%)	741	0.99 (0.94 to 1.05)	0.7
RVEF (%)	741	0.94 (0.92 to 0.95)	<0.001

D. LVGLS ≥ 16%

	N	HR (95% CI)	P value
Age (year)	398	1.04 (1.00 to 1.07)	0.054
Sex (male)	398	1.61 (0.66 to 3.93)	0.3
NYHA (Class II)	398	4.51 (1.68 to 12.1)	0.003
NYHA (Class III/IV)	398	3.89 (0.93 to 16.3)	0.063
CKD	398	1.52 (0.68 to 3.44)	0.3
LVGLS (%)	398	1.02 (0.81 to 1.27)	0.9
RVEF (%)	398	0.90 (0.86 to 0.94)	<0.001

Cardiac events: Cardiac death, HF hospitalization, ventricular tachyarrhythmia, or myocardial infarction.
Abbreviations are shown in Table S2 and S4.
