# Supplementary material for: Prognostic value of the right ventricular ejection fraction using three-dimensional echocardiography: Systematic review and meta-analysis
Source: PLoS One. 2023 Jul 7;18(7):e0287924. doi: 10.1371/journal.pone.0287924 (PMC10328342; doi:10.1371/journal.pone.0287924)
Supplement: Attachment — Submitted filename: Response to Reviewers.docx [file pone.0287924.s012.docx]

We are grateful to the editors and the reviewers for their thoughtful and constructive comments regarding our manuscript, “Prognostic Value of the Right Ventricular Ejection Fraction using Three-Dimensional Echocardiography: Systematic Review and Meta-Analysis (PONE-D-23-01475)”. Our point-by-point responses are given below in blue. Red denotes new or revised text in the manuscript.

**Additional Editor Comments:**

**I would like to thank the authors for submitting this interesting meta-analysis to PlosOne. While the meta-analysis is methodological well performed, there are some limitations concerning the clinical relevance of the findings.**

**RESPONSE:** Thank you for your positive and constructive comments.

**Major Limitations and Comments:**

**1) Reviewers’ suggestions:**

**- The reviewers have addressed important comments and suggestions that should be addressed in the meta-analysis.**

**RESPONSE:** We addressed the reviewer’s comments below.

**2) Originality and Further Analyses:**

**- Recently has been also published in JASE (Sayour et al. JASE 2023) a meta-analysis on the prognostic relevance of 3DRVEF including the same studies, with similar findings, and even showing also the incremental prognostic relevance of 3DRVEF over conventional RV parameters such as TAPSE, FAC, and RV strain. Hence, as this meta-analysis is lack of originality, further analyses should be presented to increase the clinical relevance of the findings. In this respect, the following further analyses should be performed:**

**RESPONSE:** Thank you for your comments. This study compared the prognostic value of three-dimensional (3D) right ventricular (RV) ejection fraction (EF) to left ventricular (LV) EF or LV global longitudinal strain (GLS), whereas the study by Sayour et al. compared that of RVEF to tricuspid annular plane systolic excursion (TAPSE), fractional area change (FAC) or free-wall longitudinal strain (FWLS). In this respect, we believe that the present study provides novelty and clinical relevance.

In addition, it is described that PLOS ONE editors evaluates research based on "scientific validity," "rigorous methodology," and "high ethical standards," not on whether the research is trendy or important in the eyes of a few editors (Please see below).

Taking the above into consideration, we would like you to evaluate our research once again.

We incorporated Sayour’s study in the discussion.

Page 12-13, lines 228-237:

Most recently, a meta-analysis showed that RVEF 3DE is more strongly associated with adverse outcomes than conventional echocardiography RV function parameters [15]. Ten articles (1,928 patients) reporting RVEF and conventional RV systolic parameters (TAPSE, RVFAC, or RVFWLS) on the same cohort were collected and compared for prognostic value. RVEF was robustly associated with adverse outcomes (HR: 2.64 [95% CI: 2.18 to 3.20]) and was significantly and strongly associated with adverse outcomes per 1-SD reduction compared to the other three parameters (vs. TAPSE, HR: 1.54 [95% CI: 1.04 to 2.28]; vs. RVFAC, HR: 1. 45 [95% CI: 1.15 to 1.81], vs. RVFWLS, HR: 1.44 [95% CI: 1.07 to 1.95]). However, RVEF by has not been compared to LV parameters such as LVEF or LVGLS.

Page 13, lines 243- 245:

The pooled HR in a 1-SD reduction of RVEF was 2.54 (95% CI: 2.15 - 3.00, p<0.001), showing that it is a significant predictor of adverse outcomes. The finding was in agreement with the previous meta-analysis [15].

**I) Validate the incremental prognostic value of 3DRVEF over conventional RV parameters such as TAPSE, FAC, S-TDI, and RV global and free wall strain.**

**RESPONSE:** To begin with, the purpose of this study was (1) to examine the prognostic value of RVEF and (2) to compare its prognostic value with that of LVEF or LVGLS. This study was never performed to demonstrate the validity of the study by Sayour et al.

This is for your records. Among the 15 eligible articles in our study, 10 were duplicates of the articles included in Sayour et al. Furthermore, four of the other five articles did not investigate hazard ratios (HRs) for TAPSE, FAC, or FWLS. The remaining one article by Li et al. (J Am Soc Echocardiogr. 2020;33:985-994.) reported only the HR of FAC in addition to RVEF.

Although our aim was not to validate Sayour’s study, addition of HR of FAC from only one article is not enough to validate the study by Sayour et al.

**II) Show in two further separate tables, one for PAH and another for “various CVD”, the main clinical characteristics of the studies such as sample size, feasibility of 3DRVEF, value of LVEF (2D or 3D), value of 3DRVEF, primary outcome analysis, follow-up time, and HR with 95%CI in a continuous analysis (i.e. – 1 SD) and in a dichotomous analysis (i.e. 3DRVEF < 45%).**

**RESPONSE:** Thank you for your constructive comments. We have created a table as you indicated (Table 2).

Page 8, lines 129-136:

**Table 2: Main clinical findings of studies with PAH and various CVD.**

**A: PAH**

| First author | Year | Number | Feasibility | RVEF (%) | LVEF (%) | LVGLS (%) | Primary outcome | Follow-up (months) | HR by RVEF | HR by LVEF | HR by LVGLS |
| --- | --- | --- | --- | --- | --- | --- | --- | --- | --- | --- | --- |
| Murata | 2016 | 86 | 0.87 | 43±12 | 69±8 | NA | Composite | 14.1 | 0.92 (0.88-0.96) | 1.05 (0.99-1.12) | NA |
| Moceri | 2018 | 104 | 0.92 | 35.6±9.7 | 69.1±8.5 | -8.4±3.6 | CPD | 6.6 | NA | NA | NA |
| Li | 2020 | 54 | 0.81 | 38±10 | NA | -15.4±6.8 | Composite | 28 | 0.92 (0.87-0.98) | NA | 1.27 (1.15-1.41) |
| Li | 2021 | 203 | 0.89 | 37.5±9.1 | NA | -18.6±5.5 | Composite | 20.9 | 0.90 (0.84-0.95) | NA | NA |

**B: Various CVDs**

| First author | Year | Number | Feasibility | RVEF (%) | LVEF (%) | LVGLS (%) | Primary outcome | Follow-up (months) | HR by RVEF | HR by LVEF | HR by LVGLS |
| --- | --- | --- | --- | --- | --- | --- | --- | --- | --- | --- | --- |
| Nagata | 2017 | 446 | 0.98 | 50±10 | 48±13 | -12.8±4.1 | CD | 49.2 | 0.90 (0.88-0.93) | 0.94 (0.92-0.96) | 1.23 (1.13-1.34) |
| Surkova | 2019 | 394 | 0.85 | 47±8.1 | 53.6±13.3 | -16.6±5.3 | ACD | 44.4 | 0.90 (0.88-0.92) | 0.96 (0.94-0.97) | 1.14 (1.07-1.21) |
| Muraru | 2020 | 412 | 0.63 | 47±7.4 | 53.7±13.3 | NA | Composite | 44.5 | 0.91 (0.81-0.96) | 0.93 (0.81-0.97) | NA |
| Surkova | 2021 | 292 | 0.85 | 46.5±9.2 | 49.5±14.3 | -14.7±5.1 | Composite | 80.4 | 0.94 (0.93-0.96) | 0.96(0.95-0.97) | 1.11 (1.07-1.16) |
| Kitano | 2022 | 341 | 0.98 | 46.7±11.1 | 39.7±16.3 | -15.2±4.7 | Composite | 19.8 | 0.93 (0.91-0.96) | 0.96 (0.94-0.98) | 1.12 (1.05-1.19) |

Data are expressed as number or means ± standard deviations.

CVD, cardiovascular disease; HR, hazard ratio; LVEF, left ventricular ejection fraction; LVGLS, left ventricular global longitudinal strain; PAH, pulmonary arterial hypertension; RVEF, right ventricular ejection fraction. Other abbreviations are the same as in Table 1.

**III) Concerning the analysis about the prognostic value of RVEF vs LVEF, exclude of this analysis studies with only LVEF > 50%. In addition, in the studies and analyses comparing RVEF vs LVEF, also comparing the prognostic relevance of RVEF < 45% vs LVEF < 45%.**

**RESPONSE:** Since our study is a study level meta-analysis and not an individual participant data meta-analysis, we are sorry, but we cannot perform the analysis as you suggested. In addition, we could not perform the suggested analyses because no other study reported the results of the dichotomous analysis except one study (Front Cardiovasc Med. 9:837584.).

**IV) Concerning the analysis about the prognostic value of RVEF vs GLS, comparing also the prognostic relevance of RVEF < 45% vs GLS < 16%.**

**RESPONSE:** Except for one previous study from our laboratory, no other studies have reported the results of dichotomous analysis, so we were not able to perform the analysis that you suggested.

**V) Perform a validation analysis using individual patient data from the studies of Takeuchi`s research group (PMID: 33153858 and 35282348) concerning the following analyses: feasibility of 3DRVEF, prognostic relevance of 3DRVEF < 45% and < 30% and also comparing 3DRVEF < 45% vs LVEF < 45%.**

**RESPONSE:** At first, the patients in PMID: 33153858 and PMID: 35282348 articles were overlapped. Therefore, we examined the validity of this study using data from three previous studies reported by our group (Circ Cardiovasc Imaging. 2017;10:e005384., Front Cardiovasc Med. 8:795016., Front Cardiovasc Med. 9:837584.). A total of 1,154 patients from the three studies were included, with a feasibility of 0.98 for RVEF. RVEF <45% had a significant prognostic value, while RVEF <30% did not. RVEF<45% had a comparable hazard ratio to LVEF<45%. These results were consistent with the results of this meta-analysis.

|  | Hazard ratio | 95% CI | P value |
| --- | --- | --- | --- |
| RVEF<45% | 1.32 | 1.13 – 1.54 | 0.0007 |
| RVEF<30% | 1.30 | 0.92 – 1.84 | 0.1346 |
| LVEF<45% | 1.35 | 1.15 – 1.57 | 0.0002 |

These results are only provided for the editor. We did not incorporate the results in the manuscript.

**VI) Highlight in the abstract and also in the results and discussion section that “the current medical evidence is limited on the potential prognostic relevance of 3DRVEF in patients with HFrEF and HFpEF” and “hence, further studies are warrant to determine the clinical usefulness of this potential new RV parameter in patients with HF”.**

**RESPONSE:** Thank you for the good suggestion. We have added the phrases you indicated in the discussion section.

Page 14, lines 273-275:

The current medical evidence is limited on the potential prognostic relevance of RVEF in patients with HF. Hence, further studies are warranted to determine the clinical usefulness of this potential new RV parameter in patients with HF.

**Reviewer #1:**

**Thank you for the opportunity to review the manuscript by Tetsuji Kitano et al.**

**In this thorough meta-analysis, the authors analyzed fifteen articles with 3,228 subjects. In the included studies there is some variability and the number of studies was small for a meta-analysis. Hence, these limitations should be mentioned.**

**RESPONSE:** Thank you for the comment. We have described this limitation in the study limitations section.

Page 15, lines 288-289:

Sixth, in this study, there was some variability among the included studies, and the number of studies was still small for obtaining valid conclusions.

**Reviewer #2:**

**This is a very well written manuscript. Although very interesting, I have some comments especially about the design / study population:**

**RESPONSE:** Thank you for your positive comments.

**- the study population is not clearly identified and though it’s interesting to find the RVEF is an overall excellent predictor of outcome, an explanation on the role of RV in “non RV-related” diseases is important.**

**RESPONSE:** Table 1 summarizes the characteristics of the subjects included in this study. In addition, the main clinical findings for PAH and various CVD are now summarized in Table 2

We performed a subgroup analysis, dividing PAH as RV-related diseases and various CVD as non-RV-related diseases (S1 Fig). RVEF had a prognostic value even in non-RV disease.

Supporting Information

**S1 Fig. Forest plots of hazard ratio (HR) per standard deviation (SD) reduction of right ventricular (RV) ejection fraction (EF) in patients with pulmonary arterial hypertension (upper panel) and cardiovascular disease (lower panel).**

**- it would be preferable to make the distinction within the results – “pulmonary hypertension” – “other CV diseases” – All CVD including PH**

**RESPONSE:** Thank you. We have corrected the result section as suggested.

Page 9, line 138:

All cardiovascular diseases, including pulmonary arterial hypertension

Page 9-10, lines 156-163:

Pulmonary arterial hypertension

In four studies that dealt with PAH, the pooled estimate was 2.79 (95% CI: 2.04 – 3.81, p<0.001) (S1 Fig).

Various cardiovascular diseases

In five studies investigating various cardiovascular diseases (CVD), a 1SD reduction of RVEF was associated with a 2.24-fold (95% CI: 1.77 – 2.83, p<0.001) increase in risk for adverse outcomes (S1 Fig).

**Journal requirements:**

**When submitting your revision, we need you to address these additional requirements.**

**1. Please ensure that your manuscript meets PLOS ONE's style requirements, including those for file naming. The PLOS ONE style templates can be found at**

[**https://journals.plos.org/plosone/s/file?id=wjVg/PLOSOne_formatting_sample_main_body.pdf**](https://journals.plos.org/plosone/s/file?id=wjVg/PLOSOne_formatting_sample_main_body.pdf)

**and**

[**https://journals.plos.org/plosone/s/file?id=ba62/PLOSOne_formatting_sample_title_authors_affiliations.pdf**](https://journals.plos.org/plosone/s/file?id=ba62/PLOSOne_formatting_sample_title_authors_affiliations.pdf)

**RESPONSE:** We have checked PLOS ONE's style requirements and have corrected our manuscript.

**2. Please note that PLOS ONE has specific guidelines on code sharing for submissions in which author-generated code underpins the findings in the manuscript. In these cases, all author-generated code must be made available without restrictions upon publication of the work. Please review our guidelines at https://journals.plos.org/plosone/s/materials-and-software-sharing#loc-sharing-code and ensure that your code is shared in a way that follows best practice and facilitates reproducibility and reuse.**

**RESPONSE:** Confirmed.

**3. We note that you have stated that you will provide repository information for your data at acceptance. Should your manuscript be accepted for publication, we will hold it until you provide the relevant accession numbers or DOIs necessary to access your data. If you wish to make changes to your Data Availability statement, please describe these changes in your cover letter and we will update your Data Availability statement to reflect the information you provide.**

**RESPONSE:** Confirmed.
