# Supplementary material for: Prognostic value of the right ventricular ejection fraction using three-dimensional echocardiography: Systematic review and meta-analysis
Source: PLoS One. 2023 Jul 7;18(7):e0287924. doi: 10.1371/journal.pone.0287924 (PMC10328342; doi:10.1371/journal.pone.0287924)
Supplement: Attachment — Submitted filename: Response to Reviewers.docx [file pone.0287924.s013.docx]

We are grateful to for the editor’s thoughtful and constructive comments regarding our manuscript, “Prognostic Value of the Right Ventricular Ejection Fraction using Three-Dimensional Echocardiography: Systematic Review and Meta-Analysis (PONE-D-23-01475R1)”. In accordance with the comments, we performed individual patient data analysis using three publications from our group (Nagata, Circ CVI; Nabeshima, FCVM; Kitano, FCVM). We omitted one publication (Namisaki et al., JASE 2021;34:117-26) because patients were overlapped in the publication in Kitano et al (FCVM 2022;9:837584), and Kitano’s publication had a larger number of patients. We also apologize for the delay required to revise the manuscript because we need another Ethical Review Committee approval for conducting new analyses (UOEHCRB23-023). Our point-by-point responses are given below in blue. Red denotes new or revised text in the manuscript.

**Additional Editor Comments:**

**Thank you very much for your time in revising the manuscript and for submitting your meta-analysis again to PlosOne. While some limitations have been well addressed, there are pending major limitations that should be mandatorily performed to reach acceptance in PlosOne.**

**Pending Major Limitations and Comments:**

**1) Validation analysis:**

**- The further analysis of individual patient data showing and comparing the prognostic relevance of RVEF vs. LVEF in a continuous and dichotomous analysis (i.e., RVEF < 45% vs. LVEF < 45%) using the data of the authors (Takeuchi`s research group) should be shown in a table in the results section and highlighted and discussed in the manuscript as a major finding. In addition, a separate table should show the clinical and echocardiographic characteristics of the population analyzed in this validation analysis.**

**RESPONSE:** We performed a validation analysis using individual patient data from our previous studies according to the comments. Based on the validation analysis, we revised the Materials and Methods, Results, and Discussion sections of the manuscript. The results of the validation analysis were given in Tables 3.

The analysis using individual patient data included 1,154 patients, with a mean age of 70 ± 14 years (57% male). Left ventricular (LV) ejection fraction (EF), LV global longitudinal strain (GLS), and right ventricular (RV) EF were 46.7 ± 12.6%, 13.8 ± 4.6%, and 48.1 ± 9.5%, respectively. During a median follow-up of 29.7 months, 194 patients had cardiac events. We divided patients into four groups according to 1) LVEF of 45% and RVEF of 45%, 2) LVGLS of 16% and RVEF of 45%, and performed Kaplan-Meyer survival analysis. The results are shown in new figure. We also performed univariable and multivariable Cox proportional hazard analyses. Univariable analysis revealed that not only continuous values of LVEF, LVGLS, and RVEF but also categorical values of LVEF (45% and 50%), GLS (15% and 16%), and RVEF (45%) were significantly associated with adverse outcome. Multivariable analyses showed that RVEF was significantly associated with outcome after adjusting age, sex, CKD, NYHA, and LVEF (p=0.074) and after adjusting age, sex, CKD, NYHA, and LVGLS (p=0.015). We also performed subgroup analyses in patients whose LVEF was <45% or ≥ 45% and those whose GLS was < 16% or ≥ 16%. All analyses showed that RVEF was significantly associated with adverse outcome. Finally, we performed incremental analysis. Addition of LVEF (LVGLS) had a significant incremental value over age, sex, CKD, and NYHA. Further addition of RVEF had a significant incremental value over age, sex, CKD, NYHA, and LVEF (LVGLS). All results confirmed that RVEF is a robust predictor for future outcome. We included these points in this revise manuscript.

Page 6, lines 98-102:

Validation analysis

To validate the results of this meta-analysis, a validation analysis was conducted using individual patient data from three of four previous studies in our laboratory [1, 2, 15]. One study was excluded because majority of patients were overlapped and the number of study population was smaller than the others.

Page 7, lines 124-130:

Regarding validation analysis, we performed Kaplan-Meyer survival analysis among four groups according to 1) LVEF < 45% or ≥ 45% and RVEF < 45% or ≥ 45%, and 2) LVGLS < 16% or ≥ 16% and RVEF < 45% or ≥ 45%. Next, uni- and multi-variable Cox proportional hazards regression analyses were performed. Subgroup analyses were also conducted in patients with LVEF < 45% or > 45% and LVGLS < 16% or > 16%. Finally, sequential Cox regression analysis was conducted to determine incremental value of RVEF over LVEF (LVGLS) and clinical parameters.

Page 13, lines 219-236:

Validation analysis

A total of 1,154 patients with diverse cardiovascular diseases from three publication were included. Clinical and echocardiographic characteristics were shown in Table 3. During a median follow-up of 29.7 months, 194 patients had cardiac events. Kaplan-Meier survival curve significantly stratified risk by 1) LVEF of 45% and RVEF of 45%, and 2) LVGLS of 16% and RVEF of 45% (Log-rank: p < 0.0001) (S7 Fig). Univariable Cox proportional hazard analysis revealed that not only continuous values of LVEF, LVGLS, and RVEF but also categorical values of LVEF (45% and 50%), GLS (15% and 16%), and RVEF (45%) were significantly associated with adverse outcome (Table S4 A). Multivariable analyses showed that RVEF was significantly associated with outcome after adjusting age, sex, chronic kidney disease (CKD), New York Heart Association functional class (NYHA), and LVEF (p=0.074) and after adjusting age, sex, CKD, NYHA, and LVGLS (p=0.015) (Table S4 B). Subgroup analyses in patients with LVEF < 45% or ≥ 45% and GLS < 16% or ≥ 16% also showed that RVEF was significantly associated with adverse outcomes in all analyses (Table S5). Incremental value analysis revealed that addition of LVEF (GLS) had a significant incremental prognostic value over age, sex, CKD, and NYHA. Further addition of RVEF had a significant incremental prognostic value over age, sex, CKD, NYHA, and LVEF (GLS) (S8 Fig).

Page 16, lines 300-306:

However, when we only included studies in which LVEF was impaired, the pooled ratio of HRs for RVEF and LVEF crossed 1, indicating there was no superiority of RVEF (S4 Fig). We validated the present meta-analysis using individual patient data from our three previous studies and found that RVEF had a significant incremental value over LVEF for the association with future prognosis (Table S7, S8 Fig). Therefore, loss of statistical significance may be partly related to a decrease in detection power due to a smaller number of studies (n = 7), but further validation is needed.

**- Furthermore, also using the available data of your research group (i.e., Takeuchi’s research group), please perform and compare in a second validation analysis the prognostic relevance of RVEF vs. GLS in a continuous and dichotomous analysis (i.e., RVEF < 45% vs. GLS < 16%).**

**RESPONSE:** We addressed this point in our response to the comment above.

**- These validation analyses are the main findings by which the paper will have clinical relevance given: I) the major limitations and bias of comparing RVEF vs LVEF in patients with preserved LVEF; II) the very low number of studies in patients with HFrEF or HFmrEF; and III) the lack of novelty in comparison with the meta-analysis of Sayour et al. JASE 2023 (in effect, one of the major goals of your meta-analysis was to determine the prognostic relevance of 3DRVEF (as stated in the manuscript - page 2 – paragraph 1)).**

**RESPONSE:** Thank you very much for your constructive and thoughtful comments. We understood the significance of conducting a validation analysis and we agreed with you. We added in the limitations of the manuscript that the present meta-analysis might cause significant bias in the comparison of RVEF and LVEF because many patients with preserved LVEF were included in the study.

Page 18, lines 335-337:

Fourth, the present meta-analysis might cause significant bias in the comparison of RVEF and LVEF because many patients with preserved LVEF were included in the study. In this regard, there is caution in interpreting the results, and further validation is warranted.

**2) Refine the results by excluding the studies with preserved LVEF (i.e., LVEF ≥ 50%):**

**- Including patients with preserved LVEF is a major and clinically relevant issue and limitation of the meta-analysis since it is expected and logical that LVEF will not have prognostic relevance in those patients with LVEF ≥ 50% because the values of LVEF are normal. Hence, the authors should refine the results. In this respect, the following studies (all with preserved LVEF) should be mandatorily excluded in the comparison of RVEF vs. LVEF (i.e., the studies of the following authors et al.: Murata, Moceri, Li, Li, Meng, Nabeshima (70% had LVEF ≥ 50%), Zhang, and Shen should be excluded in the comparison of the prognostic relevance of RVEF vs LVEF).**

**RESPONSE:** Thank you for your suggestions. We created a forest plot excluding the eight studies in which LVEF was preserved (S4 Fig). The pooled ratio of HRs between RVEF and LVEF was 1.34 (95% CI: 0.94 - 1.91).

As mentioned in our response to the comment #1, we also examined the prognostic value of LVEF, LVGLS, and RVEF in patients who had LVEF < 45% (n = 417) or in those whose LVGLS had < 16% (n= 741), using individual patient data. In patients with LVEF < 45%, RVEF (HR:0.95, 95% CI: 0.93 – 0.97, p < 0.001) was significantly associated with adverse outcome after adjusting age, sex, CKD, NYHA, and LVEF (HR:1.01, 95% CI: 0.99 – 1.03, p = 0.4). In patients whose LVGLS were < 16%, RVEF (HR:0.94, 95% CI: 0.92 – 0.95, p < 0.001) was significantly associated with adverse outcome after adjusting age, sex, CKD, NYHA, and LVGLS (HR:0.99, 95% CI: 0.94 – 1.05, p = 0.7). Thus, individual patient data analysis showed that RVEF had a superior predictor over LVEF (LVGLS) in patients with impaired LVEF (LVGLS). However, we think additional studies should be required to validate these results. This point mentioned in the study limitation.

Page 18, lines 335-337:

Fourth, the present meta-analysis might cause significant bias in the comparison of RVEF and LVEF because many patients with preserved LVEF were included in the study. In this regard, there is caution in interpreting the results, and further validation is warranted.

**- This major issue should also be stated and discussed in detail in the method and discussion sections, respectively.**

**RESPONSE:** We revised the Materials and Methods, Results, and Discussion section, as suggested by the editor.

Page 7, lines 124-130:

Regarding validation analysis, we performed Kaplan-Meyer survival analysis among four groups according to 1) LVEF < 45% or ≥ 45% and RVEF < 45% or ≥ 45%, and 2) LVGLS < 16% or ≥ 16% and RVEF < 45% or ≥ 45%. Next, uni- and multi-variable Cox proportional hazards regression analyses were performed. Subgroup analyses were also conducted in patients with LVEF < 45% or > 45% and LVGLS < 16% or > 16%. Finally, sequential Cox regression analysis was conducted to determine incremental value of RVEF over LVEF (LVGLS) and clinical parameters.

Page 12, lines 200-201:

When restricted to studies with reduced LVEF, the pooled ratio of HRs between RVEF and LVEF was 1.34 (95% CI: 0.94 - 1.91, p=0.092). (S4 Fig).

Page 16, lines 300-306:

However, when we only included studies in which LVEF was impaired, the pooled ratio of HRs for RVEF and LVEF crossed 1, indicating there was no superiority of RVEF (S4 Fig). We validated the present meta-analysis using individual patient data from our three previous studies and found that RVEF had a significant incremental value over LVEF for the association with future prognosis (Table S7, S8 Fig). Therefore, loss of statistical significance may be partly related to a decrease in detection power due to a smaller number of studies (n = 7), but further validation is needed.

**3) Further discussion and correction of some important sentences:**

**- Please correct and discuss with more detail the following sentences: I) “However, the prognostic usefulness of RVEF using 3DE has not been fully investigated systematically” page 3 – paragraph 2; II) “However, RVEF by has not been compared to LV parameters such as LVEF or LVGLS” page 13 – paragraph 1; III) “The current medical evidence is limited on the potential prognostic relevance of RVEF in patients with HF. Hence, further studies are warranted to determine the clinical usefulness of this potential new RV parameter in patients with HF” page 14 – paragraph 4.**

**RESPONSE:** We revised the manuscript

Page 4, lines 58-66:

Just recently, Sayour et al. conducted a systematic review and meta-analysis of the association of 3DE derived RVEF with adverse cardiopulmonary outcomes and compared the prognostic value of RVEF to TAPSE, RVFAC, and RVFWLS [13]. ~~However, the prognostic usefulness of RVEF using 3DE has not been fully investigated systematically and compared with LV parameters such as LV ejection fraction (LVEF) and LV global longitudinal strain (LVGLS).~~ However, RVEF has not been compared to LV parameters such as LVEF and LV global longitudinal strain (LVGLS), which are the most important parameters in patients with cardiovascular disease.

Page 15-16, lines 280-283:

~~However, RVEF by has not been compared to LV parameters such as LVEF or LVGLS.~~ However, RVEF has not been compared to LV parameters such as LVEF and LVGLS, which play pivotal roles in the diagnosis, treatment, and prognosis of patients with cardiovascular disease.

Page 17, lines 321-325:

The current medical evidence is limited on the potential prognostic relevance of RVEF, especially in HF patients with reduced EF. Although validation study verified that RVEF had a better predictor than LVEF or LVGLS in patients with impaired LVEF (LVEF < 45%), further meta-analyses aimed to HF patients with reduced EF are warranted to determine the clinical usefulness of 3DE determined RVEF.
