# Supplementary material for: Prognostic value of the right ventricular ejection fraction using three-dimensional echocardiography: Systematic review and meta-analysis
Source: PLoS One. 2023 Jul 7;18(7):e0287924. doi: 10.1371/journal.pone.0287924 (PMC10328342; doi:10.1371/journal.pone.0287924)
Supplement: Attachment — Submitted filename: Response to Reviewers.docx [file pone.0287924.s014.docx]

We appreciate the editor's thoughtful and constructive comments on our manuscript, “Prognostic Value of the Right Ventricular Ejection Fraction using Three-Dimensional Echocardiography: Systematic Review and Meta-Analysis (PONE-D-23-01475R2)”. Our point-by-point responses are given below in blue. Red denotes new or revised text in the manuscript.

**Journal Requirements:**

**Please review your reference list to ensure that it is complete and correct. If you have cited papers that have been retracted, please include the rationale for doing so in the manuscript text, or remove these references and replace them with relevant current references. Any changes to the reference list should be mentioned in the rebuttal letter that accompanies your revised manuscript. If you need to cite a retracted article, indicate the article’s retracted status in the References list and also include a citation and full reference for the retraction notice.**

**RESPONSE:** We have checked the list of references.

**Additional Editor Comments:**

**Thank you very much for your time and efforts in preparing and submitting your revised manuscript. Your meta-analysis, including a large and excellent validation analysis, has significantly improved and is almost ready for publication. In this regard, only minor changes remain to get the final version of this interesting and clinically relevant meta-analysis.**

**RESPONSE:** Thank you for your positive comments.

**Minor Changes:**

**- Please correct this sentence in the abstract “In subgroup analysis, HR was significantly associated with…”. Maybe you wanted to write “In subgroup analysis, RVEF was significantly associated with…”**

**RESPONSE:** Thank you for the comments. We have corrected the sentence as suggested.

Page 2, lines 27-29:

In subgroup analysis, RVEF was significantly associated with outcome in pulmonary arterial hypertension (PAH) (HR: 2.79, 95% CI: 2.04-3.82) and ~~various~~ cardiovascular (CV) diseases (HR: 2.23, 95%CI: 1.76-2.83).

**- Please edit and change this pivotal sentence in the abstract “In individual patient data analysis (n = 1,154), RVEF was significantly associated with outcome after adjusting clinical variables and LVEF or LVGLS” as “In individual patient data analysis (n = 1,142), RVEF < 45% was significantly associated with worse CV outcomes (HR 4.95 [95%CI 3.66 - 6.70]), even in patients with reduced or preserved LVEF”.**

**RESPONSE:** We have corrected the sentence as suggested.

Page 2, lines 32-35:

In individual patient data analysis (n = 1,142), RVEF < 45% was significantly associated with worse CV outcome (HR: 4.95, 95% CI: 3.66-6.70) ~~after adjusting clinical variables and LVEF or LVGLS~~, even in patients with reduced or preserved LVEF.

**- Please specify and define in the abstract, in the results section, and in each figure and table what outcomes were analyzed. By the way, an example may be “CV outcomes (HF hospitalization, mortality for HF, or CV mortality)” or “clinical and CV outcomes (mortality for all causes, HF hospitalization, mortality for HF, or CV mortality)”.**

**RESPONSE:** We have corrected as suggested.

Page 8, lines 141-144:

The primary endpoint was death (any cause, cardiac or cardiopulmonary death) in five studies and 10 other studies used a composite endpoint [all cause death, cardiac death, heart failure (HF) hospitalization, ventricular tachyarrhythmia, non-fatal myocardial infarction, PAH-related hospitalization, or PAH-related intervention].

Page 9, lines 152-153:

Composite endpoint: all cause death, cardiac death, heart failure hospitalization, ventricular tachyarrhythmia, non-fatal myocardial infarction, PAH-related hospitalization, or PAH-related intervention.

Page 10, lines 163-164:

Composite endpoint: all cause death, cardiac death, heart failure hospitalization, ventricular tachyarrhythmia, non-fatal myocardial infarction, PAH-related hospitalization, or PAH-related intervention.

Page 14, lines 231-233:

The primary endpoint was cardiac events, including cardiac death, HF hospitalization, ventricular tachyarrhythmia, or myocardial infarction.

Page 16, lines 257-258:

The primary endpoint was cardiac events, including cardiac death, HF hospitalization, ventricular tachyarrhythmia, or myocardial infarction.

Page 28, lines 519-521:

Table S4. Multivariable Cox proportional hazards analysis of (A) LVEF < 45%, (B) LVEF ≥ 45%, (C) LVGLS < 16%, (D) LVGLS ≥ 16% for cardiac events in validation study.

Table S4:

Table S4: Multivariate Cox proportional hazards analysis of (A) LVEF < 45%, (B) LVEF ≥ 45%, (C) LVGLS < 16%, (D) LVGLS ≥ 16% for cardiac events in validation study.

Cardiac events: Cardiac death, HF hospitalization, ventricular tachyarrhythmia, or myocardial infarction.

**- Please change in the abstract and in the whole manuscript the sentence/subtitle “various cardiovascular diseases” to “in patients with CV diseases”**

**RESPONSE:** We have corrected as suggested.

Page 2, lines 27-29:

In subgroup analysis, RVEF was significantly associated with outcome in pulmonary arterial hypertension (PAH) (HR: 2.79, 95% CI: 2.04-3.82) and ~~various~~ cardiovascular (CV) diseases (HR: 2.23, 95%CI: 1.76-2.83).

Page 7, lines 122-124:

Subgroup analysis was conducted according to the background disease [PAH or ~~various cardiac~~ cardiovascular (CV) disease] and modality (2DE or 3DE).

Page 10, lines 158:

Table 2: Main clinical findings of studies with PAH and ~~various~~ CV diseases.

Page 10, lines 161:

B: ~~Various~~ CV diseases

Page 11, lines 165-166:

CV~~D~~, cardiovascular ~~disease~~; HR, hazard ratio; LVEF, left ventricular ejection fraction; LVGLS, left ventricular global longitudinal strain; PAH, pulmonary arterial hypertension; RVEF, right ventricular ejection fraction.

Page 12, lines 190:

~~Various cardiovascular~~ CV diseases

Page 13, lines 191-193:

In five studies investigating ~~various cardiovascular~~ CV diseases ~~(CVD)~~, a 1-SD reduction of RVEF was associated with a 2.23-fold (95% CI: 1.76 – 2.83, p<0.001) increase in risk for adverse outcomes (S1 Fig).

Page 19, lines 324-327:

In subgroup analyses among articles, in which study subjects were solely PAH or ~~various CVDs~~ CV diseases, RVEF was significantly associated with adverse outcomes in both groups, which was consistent with previous meta-analyses in CMR-derived RVEF [20, 21].

Page 27, lines 489-490:

CI, confidence interval; CV~~D~~, cardiovascular ~~disease~~; HR, hazard ratio; PAH, pulmonary arterial hypertension; SD, standard deviation; se, standard error; TE, treatment effect.

**- Please edit and change the conclusion of the abstract to: “The findings of this meta-analysis highlight and support the use of RVEF by means of 3D transthoracic echocardiography to predict CV outcomes in routine clinical practice in patients with CV diseases and in those with PAH”.**

**RESPONSE:** We have corrected the conclusion of the abstract as suggested.

Page 3, lines 37-41:

Conclusions: ~~RVEF assessed by 3DE is significantly associated with outcome not only in PAH, but also in diverse diseases. RVEF has superior prognostic value over LVEF and similar value to LVGLS.~~ The findings of this meta-analysis highlight and support the use of RVEF assessed by 3DE to predict CV outcomes in routine clinical practice in patients with CV diseases and in those with PAH.

**- Please edit and change the conclusion in the manuscript to: “RVEF assessed by 3DE was significantly associated with adverse CV outcomes not only in patients with PAH, but also in those with CV diseases. Therefore, the findings of this meta-analysis highlight and support the use of RVEF by means of 3D transthoracic echocardiography to predict CV outcomes in routine clinical practice in patients with CV diseases and in those with PAH”.**

**RESPONSE:** We have corrected the conclusion as suggested.

Page 21-22, lines 379-386:

RVEF assessed by 3DE was significantly associated with adverse CV outcomes not only in patients with PAH, but also in those with CV diseases. ~~Furthermore, RVEF provided superior prognostic value over LVEF and similar value to LVGLS. Further studies should be performed to investigate whether a combination of both LVGLS and RVEF assessed by 3DE could effectively stratify patients with low, moderate, and high risk for future adverse outcomes.~~ Therefore, the findings of this meta-analysis highlight and support the use of RVEF assessed by 3DE to predict CV outcomes in routine clinical practice in patients with CV diseases and in those with PAH.

**- The supplemental figure 4 shows clearly that RVEF has a similar prognostic value to LVEF in studies including also patients with reduced LVEF. Likewise, supplemental table 4 also clearly shows that a reduced RVEF has similar prognostic relevance to a reduced LVEF. Hence, please state in the whole manuscript and in the abstract that “by analyzing studies including patients with reduced LVEF, RVEF had similar usefulness to LVEF to predict CV outcomes”.**

**RESPONSE:** We have added the sentence in the manuscript.

Page 20-21, lines 353-357:

Although validation study verified that RVEF had a better predictor than LVEF or LVGLS in patients with impaired LVEF (LVEF < 45%) by analyzing studies including patients with reduced LVEF, further meta-analyses focusing on HF patients with reduced EF are warranted to determine the clinical usefulness of 3DE-determined RVEF.

**- Please include in the main manuscript the excellent and clinically relevant supplemental figures s4 and s7 and the supplemental table s4.**

**RESPONSE:** We have included the S4 Fig, S7 Fig and Table S4 in the main manuscript from the supporting files.

**- In order to ensure international patient data protection and to avoid misuse of your outstanding data, please do not include in the data supplement the Excel table “supporting information / Validation analysis”.**

**RESPONSE:** Thank you for the comment. We removed the Excel file.
