# Supplementary material for: Prognostic value of the right ventricular ejection fraction using three-dimensional echocardiography: Systematic review and meta-analysis
Source: PLoS One. 2023 Jul 7;18(7):e0287924. doi: 10.1371/journal.pone.0287924 (PMC10328342; doi:10.1371/journal.pone.0287924)
Supplement: Attachment — Submitted filename: Response to Reviewers.docx [file pone.0287924.s015.docx]

We thank the editor for their careful and constructive comments on our manuscript, “Prognostic Value of the Right Ventricular Ejection Fraction using Three-Dimensional Echocardiography: Systematic Review and Meta-Analysis (PONE-D-23-01475R3)”. Our point-by-point responses are given below in blue. Red denotes new or revised text in the manuscript.

**Journal Requirements:**

**Please review your reference list to ensure that it is complete and correct. If you have cited papers that have been retracted, please include the rationale for doing so in the manuscript text, or remove these references and replace them with relevant current references. Any changes to the reference list should be mentioned in the rebuttal letter that accompanies your revised manuscript. If you need to cite a retracted article, indicate the article’s retracted status in the References list and also include a citation and full reference for the retraction notice.**

**RESPONSE:** We have checked the list of references. We did not include articles that have been retracted.

**Additional Editor Comments:**

**Thank you very much again for your time and efforts in preparing and submitting your revised manuscript. Your meta-analysis has significantly improved and is almost ready for publication. In this regard, only minor changes remain to get the final version of this clinically relevant meta-analysis.**

**RESPONSE:** Thank you for your positive comments.

**Pending Minor Changes:**

**1) Please correct the sentence in the abstract “In studies reporting HRs for both RVEF and LVEF or RVEF and LVGLS…” as “In studies reporting HRs for both RVEF and LVEF or RVEF and LVGLS in the same cohort, RVEF had 1.8-fold greater prognostic power per 1-SD reduction than LVEF (ratio of HR: 1.81, 95%CI: 1.20-2.71), but had predictive value similar to that of LVGLS (ratio of HR: 1.10, 95%CI: 0.91-1.31) and to LVEF in patients with reduced LVEF (ratio of HR: 1.34, 95%CI: 0.94 - 1.91).**

**RESPONSE:** Thank you for pointing this out. We have revised the abstract as suggested.

Page 2, lines 29-33:

In studies reporting HRs for both RVEF and LVEF or RVEF and LVGLS in the same cohort, RVEF had 1.8-fold greater prognostic power per 1-SD reduction than LVEF (ratio of HR: 1.81, 95%CI: 1.20-2.71), but had predictive value similar to that of LVGLS (ratio of HR: 1.10, 95%CI: 0.91-1.31) and to LVEF in patients with reduced LVEF (ratio of HR: 1.34, 95%CI: 0.94 - 1.91).

**2) Please add in the discussion section in the first paragraph one 6th-point: “ 6) In studies reporting HRs for both RVEF and LVEF or RVEF and LVGLS in the same cohort, RVEF had 1.8-fold greater prognostic power per 1-SD reduction than LVEF, but had predictive value similar to that of LVGLS and to LVEF in patients with reduced LVEF.**

**RESPONSE:** We have revised the discussion as suggested.

Page 17-18, lines 276-279:

(6) In studies reporting HRs for both RVEF and LVEF or RVEF and LVGLS in the same cohort, RVEF had 1.8-fold greater prognostic power per 1-SD reduction than LVEF, but had predictive value similar to that of LVGLS and to LVEF in patients with reduced LVEF.

**3) Please delete the following sentences in the discussion section because these are out of context and contradictories to the findings of the meta-analysis:**

**- “To our knowledge, this is the first meta-analysis to examine the prognostic value of RVEF using 3DE and comparing prognostic strength of RVEF with that of LVEF or LVGLS”**

**- “The current medical evidence is limited on the potential prognostic relevance of RVEF, especially in HF patients with reduced EF. Although validation study verified that RVEF had a better predictor than LVEF or LVGLS in patients with impaired LVEF (LVEF < 45%) by analyzing studies including patients with reduced LVEF, further meta-analyses focusing on HF patients with reduced EF are warranted to determine the clinical usefulness of 3DE-determined RVEF.”**

**RESPONSE:** We have revised the discussion as suggested.

Page 18, lines 279-281:

~~To our knowledge, this is the first meta-analysis to examine the prognostic value of RVEF using 3DE and comparing prognostic strength of RVEF with that of LVEF or LVGLS.~~

Page 21, lines 351-356:

~~The current medical evidence is limited on the potential prognostic relevance of RVEF, especially in HF patients with reduced EF. Although validation study verified that RVEF had a better predictor than LVEF or LVGLS in patients with impaired LVEF (LVEF < 45%) by analyzing studies including patients with reduced LVEF, further meta-analyses focusing on HF patients with reduced EF are warranted to determine the clinical usefulness of 3DE-determined RVEF.~~

**4) Please correct the figure legend of figure 6 as: Fig 6. Kaplan-Meier survival curves divided into eight groups: (A) LVEF ≥ 45% and RVEF ≥ 45%; LVEF ≥ 45% and RVEF < 45%; LVEF < 45% and RVEF ≥ 45%; LVEF < 45% and RVEF < 45%; (B) LVGLS ≥ 16% and RVEF ≥ 45%; LVGLS ≥ 16% and RVEF < 45%; LVGLS < 16% and RVEF ≥ 45%; LVGLS < 16% and RVEF < 45%.**

**RESPONSE:** We have revised the figure legend as suggested.

Page 16, lines 250-253:

Fig 6. Kaplan-Meier survival curves divided into eight groups: (A) LVEF ≥ 45% and RVEF ≥ 45%; LVEF ≥ 45% and RVEF < 45%; LVEF < 45% and RVEF ≥ 45%; LVEF < 45% and RVEF < 45%; (B) LVGLS ≥ 16% and RVEF ≥ 45%; LVGLS ≥ 16% and RVEF < 45%; LVGLS < 16% and RVEF ≥ 45%; LVGLS < 16% and RVEF < 45%.
